# Long‐term postnatal outcome of fetuses with prenatally suspected septo‐optic dysplasia

**DOI:** 10.1002/uog.22018

**Published:** 2020-09-01

**Authors:** S. Shinar, S. Blaser, D. Chitayat, T. Selvanathan, V. Chau, P. Shannon, S. Agrawal, G. Ryan, V. Pruthi, S. P. Miller, P. Krishnan, T. Van Mieghem

**Affiliations:** ^1^ Ontario Fetal Centre, Division of Maternal Fetal Medicine, Department of Obstetrics and Gynaecology, Mount Sinai Hospital University of Toronto Toronto ON Canada; ^2^ Department of Diagnostic Imaging, Hospital for Sick Children, Department of Medical Imaging University of Toronto Toronto ON Canada; ^3^ Prenatal Diagnosis and Medical Genetics Program, Department of Obstetrics and Gynecology, Mount Sinai Hospital University of Toronto Toronto ON Canada; ^4^ Division of Clinical and Metabolic Genetics, Hospital for Sick Children University of Toronto Toronto ON Canada; ^5^ Department of Paediatrics Hospital for Sick Children and University of Toronto Toronto ON Canada; ^6^ Department of Pathology and Laboratory Medicine, Mount Sinai Hospital University of Toronto Toronto ON Canada

**Keywords:** cavum septi pellucidi, CSP, septal hypoplasia, septo‐optic dysplasia, SOD

## Abstract

**Objectives:**

Septo‐optic dysplasia (SOD) is a clinical syndrome characterized by varying combinations of optic nerve hypoplasia, pituitary gland hypoplasia and abnormal cavum septi pellucidi. It is suspected on prenatal imaging when there is non‐visualization or hypoplasia of the septal leaflets. Long‐term postnatal outcomes of fetuses with prenatally suspected SOD have been documented poorly. The aims of this study were to describe the natural history of deficient septal leaflets, to quantify the incidence of postnatally confirmed SOD and to document the visual, endocrine and long‐term neurodevelopmental outcomes of these infants.

**Methods:**

This was an observational retrospective study of all fetuses with prenatal imaging showing isolated septal agenesis, assessed at a single tertiary center over an 11‐year period. Pregnancy, delivery and neonatal outcomes and pre‐ and postnatal imaging findings were reviewed. Neonatal evaluations or fetal autopsy reports were assessed for confirmation of SOD. Ophthalmologic, endocrine, genetic and long‐term developmental evaluations were assessed. Imaging findings and outcome were compared between infants with and those without postnatally confirmed SOD.

**Results:**

Of 214 fetuses presenting with septal absence on prenatal ultrasound and magnetic resonance imaging (MRI), 18 (8.4%) were classified as having suspected isolated septal agenesis suspicious for SOD. Uniform prenatal MRI findings in cases with suspected SOD included remnants of the leaflets of the cavum septi pellucidi, fused forniceal columns, normal olfactory bulbs and tracts and a normal optic chiasm. Twelve fetuses were liveborn and five (27.8%) had postnatally confirmed SOD. Only two of these five fetuses had additional prenatal imaging features (pituitary cyst, microphthalmia and optic nerve hypoplasia) supporting a diagnosis of SOD. The other three confirmed SOD cases had no predictive prenatal or postnatal imaging findings that reliably differentiated them from cases without confirmed SOD. Visual and endocrine impairments were present in two (40%) and four (80%) cases with confirmed SOD, respectively. In those with visual and/or endocrine impairment, developmental delay (median age at follow‐up, 2.5 (interquartile range, 2.5–7.0) years) was common (80%) and mostly severe. Neonates with isolated septal agenesis and a lack of visual or endocrine abnormalities to confirm SOD had normal development.

**Conclusions:**

Only a quarter of fetuses with isolated septal agenesis suggestive of SOD will have postnatal confirmation of the diagnosis. Clinical manifestations of SOD are variable, but neurodevelopmental delay may be more prevalent than thought formerly. © 2020 Authors. *Ultrasound in Obstetrics & Gynecology* published by John Wiley & Sons Ltd on behalf of International Society of Ultrasound in Obstetrics and Gynecology.

**CONTRIBUTION**

*What are the novel findings of this work?*

A quarter of fetuses with isolated septal agenesis have septo‐optic dysplasia (SOD). Magnetic resonance imaging findings suspicious for SOD in fetuses with isolated septal agenesis include septal remnants and fused forniceal columns. Ocular and pituitary findings are supportive, but do not distinguish between infants with and those without SOD. Developmental delay is more prevalent in infants with SOD than reported previously.

*What are the clinical implications of this work?*

Although SOD cannot be diagnosed prenatally, we present prenatal imaging findings that are suggestive of the condition in fetuses with isolated septal agenesis. We provide long‐term outcome data on fetuses with hypoplasia of the septal leaflets, according to whether they were diagnosed with SOD. This should help in prenatal counseling of patients.

## INTRODUCTION

Septo‐optic dysplasia (SOD), also known as De Morsier syndrome, is a rare congenital disorder, affecting 1 in 10 000 live births^1^, ^2^. The condition is defined as the co‐occurrence of two or more of the following three diagnostic features: optic nerve hypoplasia, pituitary hypoplasia and septal leaflet abnormality^3^, ^4^, ^5^. Prenatally, SOD is usually suspected when there is absence of the cavum septi pellucidi due to septal leaflet deficiency, while more specific features, including the presence of septal remnants and fused forniceal columns, are confirmed on magnetic resonance imaging (MRI)^3^. The final diagnosis, however, can only be confirmed postnatally, when pituitary function tests and ophthalmologic assessments are performed. SOD is a clinically heterogeneous syndrome with approximately only one‐third of all affected individuals demonstrating all three diagnostic features^6^. Neurodevelopmental delay and behavioral disorders, though not diagnostic, are also common in this syndrome^7^.

Prenatally, SOD is suspected when there is septal leaflet hypo‐ or dysgenesis, leading to lack of a visualized cavum septi pellucidi. However, the differential diagnosis of septal leaflet absence is broad and other brain anomalies should also be considered, including primary brain disorders, such as holoprosencephaly (with septal agenesis), agenesis/dysgenesis of the corpus callosum (with widely separated or malpositioned septal leaflets^8^), ballooning of the cavum septi pellucidi (with lateral displacement of the septal leaflets, as has been reported in 22q11 microdeletion syndrome^9^), hydrocephalus (with medial leaflet displacement and a small or collapsed cavum), as well as secondary disruptive or compressive processes (hydrocephalus, hydranencephaly and schizencephaly/porencephaly)^10^, ^11^. Isolated absence of the septal leaflets has also been described as a normal variant^12^. Imaging can assist in distinguishing between the different etiologies: MRI and ultrasound can be used to demonstrate supportive findings, such as hypoplasia of the optic chiasm and optic nerves^13^, ^14^, ^15^ or a hypoplastic pituitary gland. Vision and endocrine function, however, can be assessed definitively only postnatally.

Prenatal counseling parents of a fetus with suspected SOD, on the basis of non‐visualized septal leaflets, is particularly challenging, as the literature on the long‐term outcome of prenatally suspected SOD is very scarce. Postnatal series have documented a high incidence of developmental delay, visual problems and pituitary dysfunction in children with SOD^16^, ^17^, but these series may be biased by the fact that infants diagnosed postnatally are typically only assessed due to symptoms. Cases suspected prenatally may therefore have a more benign course and heterogeneous expression, particularly if the diagnosis is not confirmed postnatally.

The aims of this study were to describe the natural history of prenatally suspected SOD, to quantify the incidence of postnatally confirmed SOD and to document the visual, endocrine and long‐term neurodevelopmental outcomes of these infants.

## METHODS

Following approval of the study protocol by the Research Ethics Boards at Mount Sinai Hospital, Toronto, ON, Canada (REB #18‐0004‐C) and at the Hospital for Sick Children, Toronto, ON, Canada (REB #1000063300), we conducted an observational retrospective cohort study of all fetuses with prenatally suspected SOD. We reviewed the charts of all fetuses that presented with non‐visualized septal leaflets on ultrasound, at the Ontario Fetal Centre, Mount Sinai Hospital, between 1^st^ January 2008 and 30^th^ June 2019. The diagnosis was confirmed by fetal MRI in most cases. To ensure that we captured all cases with prenatally suspected SOD, we also reviewed all fetal MRI examinations demonstrating fused forniceal columns and all fetal autopsies revealing dehiscent, hypoplastic or ruptured septal leaflets. Cases in which other major central nervous system anomalies were seen, or in which imaging features confirmed a different etiology for the absent cavum septi pellucidi, are summarized in Table 1 and were excluded from further analyses. The remaining cases were classified as having prenatally suspected isolated SOD and comprised the study cohort.

**Table 1:** Suspected prenatal diagnosis in 214 fetuses with absent cavum septi pellucidi

Suspected diagnosis	*n* (%)
Septo‐optic dysplasia	18 (8.4)
Anomaly of corpus callosum*	84 (39.3)
Severe ventriculomegaly†	33 (15.4)
Aqueductal stenosis	32 (15.0)
Holoprosencephaly	21 (9.8)
Neural tube defect‡	16 (7.5)
Porencephalic cyst	4 (1.9)
Cortical malformation§	3 (1.4)
Syntelencephaly	3 (1.4)
Hydranencephaly	1 (0.5)
Intraventricular hemorrhage without severe ventriculomegaly	1 (0.5)

* Included agenesis, partial agenesis, hypoplasia and dysplasia.

† Defined as atrial width of lateral ventricles ≥ 15 mm.

‡ Two fetuses also had agenesis of corpus callosum.

§ Included schizencephaly, lissencephaly and polymicrogyria.

Ultrasound examinations were performed transabdominally using a Philips iU‐22 (Philips Healthcare, PA, USA) or Voluson E10 (GE Healthcare, Zipf, Austria) ultrasound machine and, when required, were complemented by a transvaginal examination. Prenatal and postnatal MRI examinations were performed using a 1.5‐T Twinspeed imaging system (GE Healthcare, Milwaukee, WI, USA), utilizing multiplanar single‐shot fast‐spin echo.

For fetuses meeting the inclusion criteria, pregnancy and delivery outcomes were retrieved. For liveborn infants, neonatal evaluations were reviewed for clinical confirmation of SOD. Postnatal outcomes were analyzed from the ophthalmologic, endocrine, developmental and genetics follow‐up clinics at Mount Sinai Hospital and the Hospital for Sick Children. Specifically, pituitary function tests, visual acuity, fundoscopy and long‐term motor, verbal and cognitive outcomes were assessed. Developmental assessment was completed according to standardized developmental tests. For cases in which the parents opted for pregnancy termination, fetal autopsy reports were reviewed, when available, for confirmation of absent, hypoplastic, ruptured or dehiscent septal leaflets and postmortem diagnosis.

## RESULTS

Over the study period, 214 fetuses with absent septal leaflets on ultrasound were identified (Table 1). After exclusion of cases with other etiologies, 18 (8.4%) cases were classified as having prenatally suspected isolated SOD. Maternal, obstetric and neonatal characteristics of these cases are shown in Table 2. In one case, a previous child of the same parents was also diagnosed with absent septal leaflets; no clear genetic etiology was identified. Prenatal genetic testing was carried out in nine patients; microarray was normal in eight and demonstrated a variant of unknown significance in chromosome 10p13 in one, who was diagnosed postnatally with SOD.

**Table 2:** Maternal, obstetric and neonatal characteristics in 18 pregnancies with fetal isolated hypoplastic septal leaflets, overall and according to whether septo‐optic dysplasia (SOD) was confirmed postnatally

		Postnatal diagnosis	
Parameter	Prenatally suspected SOD (*n* = 18)	SOD (*n* = 5)	Non‐SOD (*n* = 13)
Maternal age (years)	31.3 ± 6.7	27.2 ± 6.8	33.0 ± 6.2
Nulliparous	10 (55.6)	3 (60.0)	7 (53.8)
Diabetes	2 (11.1)	1 (20.0)	1 (7.7)
Hypertension	2 (11.1)	1 (20.0)	1 (7.7)
Assisted reproduction	1 (5.6)	0 (0)	1 (7.7)
NT > 3 mm	0/12 (0)	0/4 (0)	0/8 (0)
High risk on eFTS	2/12 (20.0)	1/4 (25.0)	1/8 (12.5)
Abnormal NIPT	0/2 (0)	0/1 (0)	0/1 (0)
Normal karyotype/CMA	8/9 (88.9)†	4 (80.0)†	4/4 (100)
Gestational age at diagnosis (weeks)	24.5 ± 4.7	25.3 ± 4.7	24.3 ± 5.8
Termination of pregnancy	4 (22.2)	0 (0)	4 (30.8)
Gestational age at delivery (weeks)*	36.3 ± 5.3	36.8 ± 3.7	35.9 ± 6.1
Induction of labor*	2/14 (14.3)	0/5 (0)	2/9 (22.2)
Cesarean delivery	4 (22.2)	1 (20.0)	3 (23.1)
Live birth	12 (66.7)	5 (100)	7 (53.8)
Stillbirth	2 (11.1)	0 (0)	2 (15.4)
Female sex	8 (44.4)	3 (60.0)	5 (38.5)
Birth weight (g)*	2546 ± 1095	2690 ± 978	2444 ± 1238
Birth weight < 5^th^ percentile*	1/14 (7.1)	0/5 (0)	1/9 (11.1)
5‐min Apgar score	9 (7–9)	9 (8–9)	9 (6–9)

Data are given as mean ± SD, *n* (%), *n*/*N* (%) or median (interquartile range).

* Cases of termination of pregnancy excluded.

† One patient had variant of unknown significance in chromosome 10p13 of 0.019 MB.

CMA, chromosomal microarray analysis; eFTS, enhanced first‐trimester screening; NIPT, non‐invasive prenatal testing; NT, nuchal translucency.

**Figure 1:**
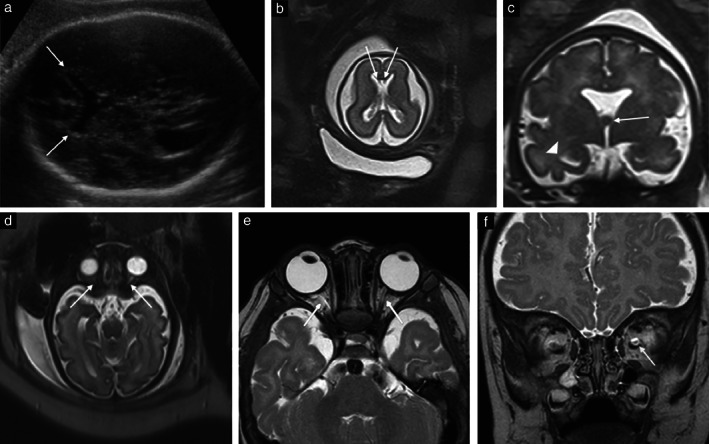
Prenatal and postnatal imaging in fetuses with isolated septal agenesis that had postnatally confirmed septo‐optic dysplasia. (a) Axial ultrasound image at 31 + 1 weeks' gestation, demonstrating fused anterior horns, absence of cavum septi pellucidi and squared anterior horns (arrows). (b) T2‐weighted axial magnetic resonance (MR) image at 23 + 4 weeks, demonstrating fused anterior horns and septal remnants (arrows). (c) T2‐weighted coronal MR image at 34 + 0 weeks, demonstrating fused forniceal columns (arrow) and dysplastic temporal horns (arrowhead). (d) T2‐weighted axial MR image at 33 + 6 weeks, demonstrating bilaterally hypoplastic optic nerves (arrows). (e) T2‐weighted axial MR image at 4 months of age, demonstrating hypoplastic extra‐orbital optic nerves (arrows). (f) T2‐weighted coronal MR image at 4 months of age, demonstrating hypoplastic nerve within fluid‐filled optic nerve sheath (arrow).

Twelve (66.7%) patients with suspected isolated septal agenesis opted to continue the pregnancy and delivered a liveborn neonate. Of these, 11 underwent fetal MRI (Table 3), which demonstrated remnants of the leaflets of the cavum septi pellucidi, fused forniceal columns, normal olfactory bulbs and tracts and a normal optic chiasm in all cases (Figure 1). Six fetuses also had unilateral or bilateral mild ventriculomegaly (ventricular atrial width, 10–12 mm) and one had unilateral moderate ventriculomegaly (ventricular atrial width, 12.7 mm). Optic nerves were present in all fetuses, but appeared bilaterally hypoplastic in one (Figure 1). This fetus also presented with bilateral mild ventriculomegaly. The pituitary gland was of normal size and appearance in all but one case, in which a cyst measuring 8.8 × 10.1 mm (midline teratoma or Rathke cleft cyst) was visualized. In this fetus, there was also unilateral microphthalmia and mild unilateral ventriculomegaly. Nine neonates underwent postnatal brain MRI examination, while one had only postnatal ultrasound examination. Postnatal MRI confirmed a pituitary lesion and microphthalmia in one neonate and also demonstrated unilateral optic nerve hypoplasia of the affected eye. Additional findings diagnosed postnatally included a hypoplastic splenium of the corpus callosum in one neonate and a thinned corpus callosum in another. In one neonate, the corpus callosum was described as thinned prenatally, secondary to bilateral mild ventriculomegaly, but had normal thickness postnatally.

**Table 3:** Prenatal and postnatal imaging findings in 12 neonates with prenatally suspected septo‐optic dysplasia

Imaging finding	Value
Ultrasound	12 (100)
Absent cavum septi pellucidi	12 (100)
Mild ventriculomegaly*	6 (50.0)
Moderate ventriculomegaly†	1 (8.3)
Additional findings	5 (41.6)
Echogenic cardiac focus	1 (8.3)
Oligohydramnios‡	2 (16.7)
Hypoplastic nasal bone	1 (8.3)
Retromicrognathia	1 (8.3)
Prenatal MRI	11 (91.7)
Septal remnants	11/11 (100)
Forniceal fusion	11/11 (100)
Thinned corpus callosum	4/11 (36.4)
Squared anterior horns	8/11 (72.7)
Dysplastic temporal horns	8/11 (72.7)
Olfactory bulbs present	11/11 (100)
Hypoplastic optic chiasm	0/11 (0)
Hypoplastic optic nerves	1/11 (9.1)
Unilateral microphthalmia	1/11 (9.1)
Abnormal pituitary gland	1/11 (9.1)
Mild‐to‐moderate ventriculomegaly	7/11 (63.6)
Postnatal ultrasound/MRI	10 (83.3)
Age at scan (days)	3.5 ± 1.7
Concordant findings pre‐ and postnatally	6/10 (60.0)
Non‐concordant or additional findings	4/10 (40.0)

Data are given as *n* (%), *n*/*N* (%) or mean ± SD.

* Defined as atrial width of lateral ventricles of 10–12 mm.

† Defined as atrial width of lateral ventricles of 12.1–14.9 mm.

‡ Both cases were complicated by preterm prelabor rupture of membranes.

MRI, magnetic resonance imaging.

Clinical outcome, including endocrine, ophthalmologic and long‐term neurodevelopmental assessments, were available for 10 infants (Table 4). Two patients were followed to term, but were lost to postnatal follow‐up. Median age at developmental follow up was 3 years and 4.5 years for patients with postnatally confirmed SOD and those without SOD, respectively. Five (50%) patients fulfilled criteria for the diagnosis of SOD, with one meeting all three diagnostic criteria and four meeting two. Two patients had abnormal ophthalmologic examinations. Of these, one had left‐sided microphthalmia on pre‐ and postnatal imaging, and unilateral corneal clouding and a unilateral hypoplastic optic nerve on postnatal imaging. This patient also had a pituitary cyst. The other patient demonstrated bilateral optic nerve hypoplasia (Figure 1) on pre‐ and postnatal imaging, with involvement of the optic chiasm and bilateral nystagmus. Both of these patients had impaired vision. They also demonstrated mild ventriculomegaly. Ventriculomegaly was a common finding both pre‐ and postnatally, seen in four of the five patients with SOD. Four patients had failure to thrive (defined as weight and/or height below the 3^rd^ percentile) and were persistently below the 3^rd^ percentile for weight, of whom two were also below the 10^th^ percentile for height on all endocrine assessments. Postnatal insulin‐like growth factor‐1 level was available in three patients and was within normal range in all. Sodium, glucose and morning cortisol levels were normal in all 10 patients. Four patients demonstrated high thyroid‐stimulating hormone, which normalized on subsequent assessments. There were no cases of precocious puberty.

**Table 4:** Clinical short‐ and long‐term outcomes of 10 fetuses with suspected septo‐optic dysplasia (SOD), according to whether diagnosis was confirmed postnatally

Outcome	SOD (*n* = 5)	Non‐SOD (*n* = 5)
GA at delivery (weeks)	36.8 ± 3.7	36.3 ± 5.7
Birth weight (g)	2690 ± 978	3134 ± 440
Normal karyotype/CMA	4 (80.0)†	4/4 (100)
Age at final assessment (years)	3 (3–7)	4.5 (2.5–8)
Age at last ophthalmologic examination (weeks)	16 (6–27)	0.2 (0.1–0.4)
Abnormal ophthalmologic examination	2 (40.0)	0 (0)
Age at last endocrine assessment (days)	2.25 (1–2.5)	2.25 (1–2.5)
Abnormal pituitary function tests	0 (0)	0 (0)
Failure to thrive*	4 (80.0)	0 (0)
Age at last developmental assessment (years)	2.5 (2.5–7.0)	2.5 (1.75–2.5)
Developmental delay	4 (80.0)	0/4 (0)
Verbal	4 (80.0)	0/4 (0)
Motor	3 (60.0)	0/4 (0)
Cognitive	3 (60.0)	0/4 (0)
Seizures	2 (40.0)	0/4 (0)

Data are given as mean ± SD, *n* (%), median (interquartile range) or *n*/*N* (%).

* Defined as weight and/or height < 3^rd^ percentile.

†One patient had variant of unknown significance in chromosome 10p13 of 0.019 MB.

CMA, chromosomal microarray analysis; GA, gestational age.

Developmental assessments were available in all patients with confirmed SOD and in four of the five non‐SOD patients. Developmental assessments were abnormal in four children, all of whom had SOD. Three patients demonstrated global developmental delay, of whom two also had seizures. All three of these patients also had failure to thrive, but no additional endocrine abnormality. One child had a verbal delay and hypotonia. All four patients with deficient septal leaflets, but no postnatal confirmation of SOD, who had complete postnatal follow‐up, had normal development, with the last assessment performed at a median of 2.5 (interquartile range, 1.75–2.5) years of age.

**Figure 2:**
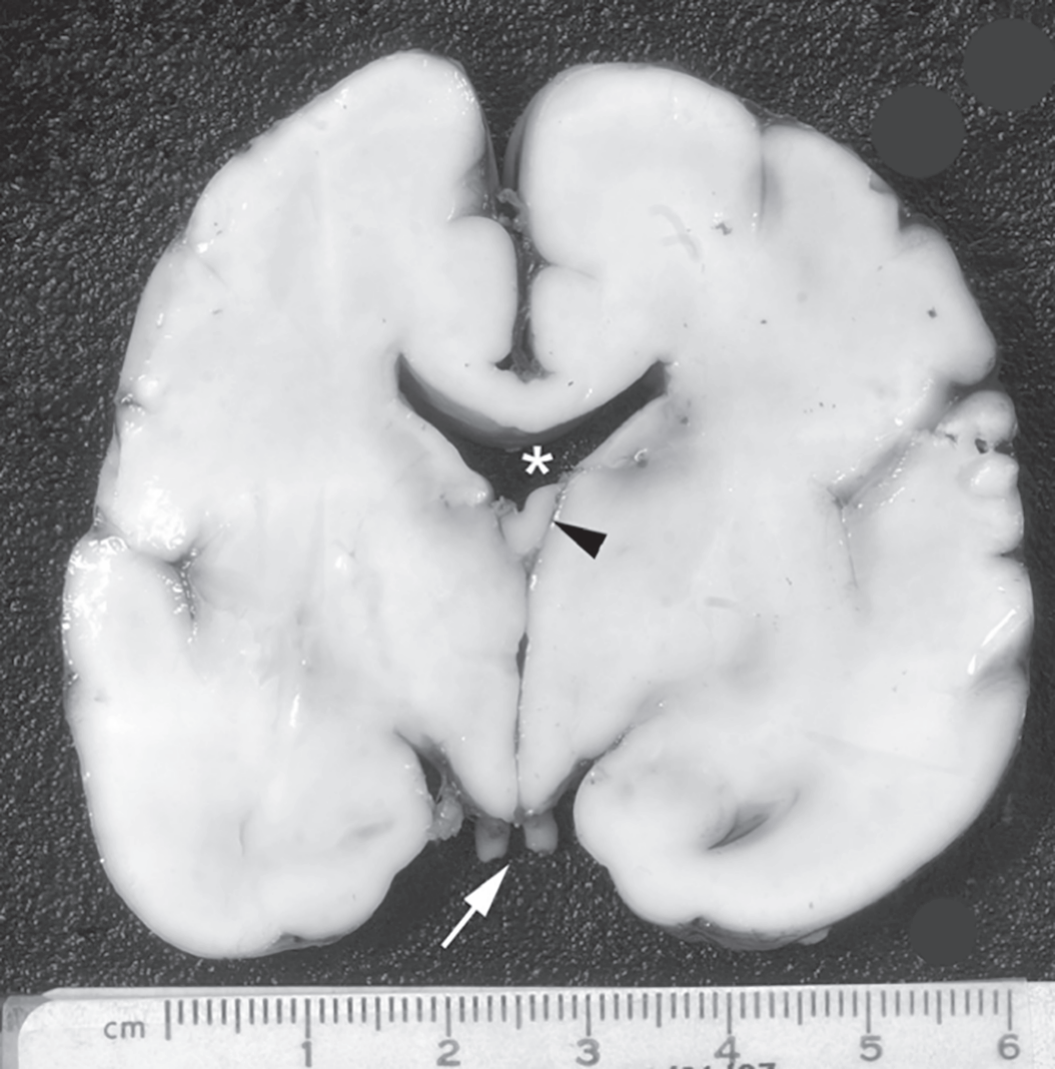
Coronal section of brain of stillborn fetus diagnosed prenatally with isolated septal agenesis at 31 weeks of gestation. Septal leaflets were absent (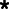) and fornices were fused and low‐lying (arrowhead). Ventricular contour was abnormal, although dilated only minimally. Optic nerves (arrow) were normal.

In four of 18 patients with suspected SOD, the parents opted to terminate the pregnancy and fetal autopsy was performed. Two cases in which pregnancy was continued were complicated by preterm prelabor rupture of membranes at 18 and 22 weeks' gestation and subsequently had preterm labor and stillbirth at 30 and 23 weeks, respectively. Autopsy was performed in both of these cases. All six autopsies demonstrated disrupted septal remnants, of which five were secondary to disruptive processes (aqueductal dysplasia (*n* = 1), aqueductal stenosis (*n* = 1) and intraventricular hemorrhage and ventriculomegaly (*n* = 3)) and one was a primary agenesis of the septum, with normal ventricular atrial width and bifrontal polymicrogyria, which was not detected on prenatal ultrasound or MRI. In all autopsy cases, the optic nerve, chiasm and pituitary gland were normal in size and appearance (Figure 2).

## DISCUSSION

We present a large single‐center experience of postnatal outcome of fetuses with suspected SOD, isolated from a cohort of over 200 fetuses with septal leaflet deficiency. As demonstrated previously^7^, ^11^, ^18^, ^19^, clinical SOD was confirmed postnatally in only a minority of the cases. MRI findings in prenatally suspected cases included remnants of the septal leaflets of the cavum septi pellucidi, fused forniceal columns, normal olfactory bulbs and tracts and a normal optic chiasm. There were two patients with a higher suspicion of SOD based on prenatal imaging, of which one had a pituitary cyst and the other had optic nerve hypoplasia. Nonetheless, no prenatal sonographic or MRI findings were predictive of SOD. Thus, the importance of these findings is in their sensitivity, and not specificity, for the diagnosis of SOD. Prenatal suspicion is critical, as the diagnosis of SOD may otherwise be delayed if visual and pituitary dysfunction are not evident early in life. Fetuses with septal leaflet deficiency, but not SOD, had normal outcome. In contrast, patients with SOD had a highly variable disease spectrum, and four of five had significant developmental delay. This wide outcome spectrum, from completely normal to severely delayed, underlines the difficulty in prenatal counseling of couples expecting a fetus with fused anterior horns associated with absent cavum septi pellucidi, due to deficient septal leaflets.

Five other small case series have reported previously on the postnatal outcome of prenatally suspected SOD^7^, ^11^, ^18^, ^19^, ^20^ (Table 5). In line with the results from these studies, we found that the majority of patients with confirmed SOD manifest the typical brain findings with either visual problems or partial pituitary dysfunction, but usually not both. The association between hypoplastic septal leaflets and pituitary deficiencies is not surprising, given that they are both midline structures. However, it should be noted that children with optic nerve hypoplasia and normal head imaging can still manifest endocrinopathies^21^. Unlike previous studies, which showed rather reassuring findings with regards to neurodevelopment^7^, ^18^, ^19^, we found a high incidence of developmental delay, mostly global and severe. This difference could potentially be explained by the fact that two of the other studies did not obtain long‐term follow‐up of sufficient duration to assess adequately the incidence of developmental delay^11^, ^19^ and some studies had incomplete neurological follow‐up for a relatively large number of patients^18^. The high rates of developmental delay observed in our series may also be associated with visual impairment, particularly when severe, and with pituitary disease. This is highlighted by the finding that all individuals with global developmental delay also exhibited failure to thrive.

**Table 5:** Summary of case series reporting outcome of fetuses with prenatally suspected septo‐optic dysplasia (SOD), according to whether diagnosis was confirmed

			Non‐SOD		Confirmed SOD					
					
Study	Prenatally suspected SOD	No additional major brain anomalies	Cases	Abnormal neuro‐development	Cases	Abnormal ophthalmologic exam	Pituitary dysfunction	Abnormal neuro‐development	Follow‐up length (years)	Lost to follow‐up
Lepinard (2005)^20^	2	2	1	0	1	1	1	1	2.1 (1.2–3.1)	0
Malinger (2005)^11^	2	0	1	0	0	0	0	0	0.5	0
Damaj (2010)^7^	17	17	13	4	3	2	1	0	3 (1.8–3.8)	1
Pilliod (2018)^18^	15	13	6	2	2	2	1	0	2.5 (2–3)	7
Vawter‐Lee (2018)^19^	8	8	6	1	2	1	1	0	0.7 (0.7–0.8)	0
Present study	18	18	5	0	5	2	4	4	2.5 (2.5–7)	2
Total	62	58 (93.5)	32 (51.6)	7 (21.9)	13 (21.0)	8 (61.5)	8 (61.5)	5 (38.5)	—	10 (16.1)

Only first author of each study is given. Data are given as *n*, *n* (%) or median (interquartile range). Case reports not included. Total number of cases of confirmed SOD and non‐SOD may not equal number of cases of prenatally suspected SOD due to patients lost to follow‐up and termination of pregnancy.

Since a clinical diagnosis of SOD can be fully established only after birth, previous studies assessing outcomes of SOD were done primarily in pediatric and adult populations^10^, ^16^, ^17^, ^22^, ^23^. These studies also showed a high incidence of developmental delay (up to 78%), but did not necessarily differentiate between isolated SOD and SOD with cortical malformations^17^. In one of the larger studies, all 18 patients with SOD had poor vision or blindness, even though only 82% had optic nerve hypoplasia. Partial or complete pituitary dysfunction has been reported to complicate up to 78% of cases of SOD^23^, with the most common deficiencies being in growth and central hypothyroidism. A gradual decrease in hormone secretion was an important characteristic of SOD in that series and thus, periodic evaluations are recommended even if no endocrinological problems are evident at birth^23^. While short stature and failure to thrive were common in our series, despite normal growth hormone levels, poor vision, with or without optic nerve hypoplasia, was not. The reported higher incidence of optic nerve hypoplasia and visual disturbance in individuals with SOD in previous studies may stem from the fact that, by definition, all our patients had deficient septal leaflets. Thus, fetuses with optic nerve hypoplasia and pituitary dysfunction, but an intact septum, would not be captured in this prenatal series, but would still be defined as having SOD. This hypothesis is supported by the known increased risk for hypothalamic‐pituitary dysfunction seen in approximately 60% to 80% of individuals with optic nerve hypoplasia^24^. The difference in the spectrum of prenatally suspected SOD *vs* pediatric or adult SOD can be explained by the fact that patients in the latter group are typically assessed only due to symptoms consistent with the diagnosis of SOD. Thus, the lower incidence and milder forms of pituitary and ophthalmic disease in this series is not surprising.

The strengths of this study lie in the systematic long‐term follow‐up of the patient cohort, with comprehensive assessments, including dedicated neurodevelopmental assessments. Moreover, all cases underwent detailed fetal ultrasound and brain MRI examinations, which enabled accurate diagnosis. Finally, our strict inclusion criteria excluded cases with significant additional brain anomalies or hydrocephalus impacting prognosis, thereby allowing us to focus on outcomes related directly to isolated SOD. The retrospective nature of the study, however, introduces some limitations. First, two cases were lost to postnatal follow‐up. Second, the utilization of a 1.5‐T imaging system may be suboptimal for prenatal assessment of the optic nerves. Third, genetic testing was limited to the routine clinical strategies. Next‐generation sequencing is now available and may be able to shed further light on new genetic causes^25^, ^26^. Fourth, our postnatal follow‐up was limited in time and patients were not followed to puberty. As a result, we were unable to assess fully for hypogonadotropic hypogonadism secondary to luteinizing hormone and follicle‐stimulating hormone deficiency, which is a possible manifestation of SOD. Lastly, the single‐center nature of this study may limit the generalizability of the results. It is possible that, in general practice, non‐visualization or hypoplasia of the septal leaflets is suspected prenatally with greater variability in methods and, thus, potentially even lower accuracy.

In conclusion, prenatal counseling in cases of fetal isolated absent septal leaflets remains challenging. In this study, we provided data from a large single‐center cohort, and summarized previously published case series to obtain more reliable statistics, with which to counsel patients expecting a baby with an absent cavum septi pellucidi, due to deficient septal leaflets. Though no prenatal findings are diagnostic of SOD, ventriculomegaly and ocular and pituitary findings are supportive, but their absence does not reliably exclude this condition. Approximately a quarter of neonates with prenatally suspected SOD will have postnatally confirmed SOD, with varying degrees and severity of visual, pituitary and developmental dysfunction. Future studies with longer follow‐up into puberty are required to better ascertain the spectrum of pituitary dysfunction and neurodevelopmental delay in SOD.
